# Lipid Deposition in Skeletal Muscle Tissues and Its Correlation with Intra-Abdominal Fat: A Pilot Investigation in Type 2 Diabetes Mellitus

**DOI:** 10.3390/metabo15010025

**Published:** 2025-01-07

**Authors:** Manoj Kumar Sarma, Andres Saucedo, Suresh Anand Sadananthan, Christine Hema Darwin, Ely Richard Felker, Steve Raman, S. Sendhil Velan, Michael Albert Thomas

**Affiliations:** 1 Radiological Sciences, David Geffen School of Medicine at UCLA, Los Angeles, CA 90095, USA; manojkumar.sarma@utsouthwestern.edu (M.K.S.); efelker@mednet.ucla.edu (E.R.F.); sraman@mednet.ucla.edu (S.R.); 2 Institute for Human Development and Potential, Agency for Science, Technology, and Research (A*STAR), Singapore 117609, Singapore; suresh@sics.a-star.edu.sg (S.A.S.); sendhil_velan@sics.a-star.edu.sg (S.S.V.); 3 Medicine, David Geffen School of Medicine at UCLA, Los Angeles, CA 90095, USA; cdarwin@mednet.ucla.edu

**Keywords:** MR spectroscopic imaging, calf muscle lipids and metabolites, intra-abdominal fat, type 2 diabetes mellitus

## Abstract

**Background/Objectives:** This study evaluated metabolites and lipid composition in the calf muscles of Type 2 diabetes mellitus (T2DM) patients and age-matched healthy controls using multi-dimensional MR spectroscopic imaging. We also explored the association between muscle metabolites, lipids, and intra-abdominal fat in T2DM. **Methods:** Participants included 12 T2DM patients (60.3 ± 8.6 years), 9 age-matched healthy controls (AMHC) (60.9 ± 7.8 years), and 10 young healthy controls (YHC) (28.3 ± 1.8 years). We acquired the 2D MR spectra of calf muscles using an enhanced accelerated 5D echo-planar correlated spectroscopic imaging (EP-COSI) technique and abdominal MRI with breath-hold 6-point Dixon sequence. **Results:** In YHC, choline levels were lower in the gastrocnemius (GAS) and soleus (SOL) muscles but higher in the tibialis anterior (TA) compared to AMHC. YHC also showed a higher unsaturation index (U.I.) of extramyocellular lipids (EMCL) in TA, intramyocellular lipids (IMCL) in GAS, carnosine in SOL, and taurine and creatine in TA. T2DM patients exhibited higher choline in TA and myo-inositol in SOL than AMHC, while triglyceride fat (TGFR2) levels in TA were lower. Correlation analyses indicated associations between IMCL U.I. and various metabolites in muscles with liver, pancreas, and abdominal fat estimates in T2DM. **Conclusions:** This study highlights distinct muscle metabolite and lipid composition patterns across YHC, AMHC, and T2DM subjects. Associations between IMCL U.I. and abdominal fat depots underscore the interplay between muscle metabolism and adiposity in T2DM. These findings provide new insights into metabolic changes in T2DM and emphasize the utility of advanced MR spectroscopic imaging in characterizing muscle-lipid interactions.

## 1. Introduction

Type 2 diabetes (T2DM) is a chronic metabolic condition where every aspect of the body’s metabolism is altered [1]. Insulin resistance (IR) is a common feature of T2DM and an early metabolic abnormality [2]. IR is considered a pivotal element of the metabolic syndrome mainly because of its substantial impact on liver and muscle [3]. It is known that lipids are stored as triglycerides in the adipose tissues or in lipid droplets in the cytoplasm of non-adipose cells. IR leads to impaired glucose absorption in muscle cells; manifests a serious breakdown in lipid dynamics; and promotes the hydrolysis of triglycerides, the release of free fatty acids (FA), the reduced esterification and re-esterification of FA in adipose tissues (ATs), and hepatic gluconeogenesis [4,5]. This causes an abnormal buildup of lipids in non-adipose tissues, including skeletal muscle, abdominal visceral and hepatic fat depots, and pancreatic beta cells [6,7], along with macrophage infiltration and inflammation [8]. This fat deposition in non-adipose tissues leads to further IR [9] and oxidative stress, and it has deleterious effects, including tissue damage (lipotoxicity) [10]. Skeletal muscle represents the primary site for postprandial glucose disposal [11]. Two pools of lipids are found in skeletal muscle tissues: intra-myocellular lipids (IMCLs), stored as spherical droplets within muscle cells adjacent to mitochondria, and extra-myocellular lipids (EMCLs), distributed over large regions of muscle fascia. There is also a large interest in understanding the fat composition within these lipid pools. The influence of fatty acids on metabolic signaling and energy metabolism is affected by their level of unsaturation [12]. Monounsaturated and polyunsaturated fatty acids are produced through the desaturation of saturated fatty acids via an oxidative reaction catalyzed by stearoyl CoA desaturase, an iron-containing microsomal enzyme. Consequently, this enzyme regulates the degree of unsaturation, and its activity significantly impacts lipid metabolism [12]. Recent evidence suggests that the accretion of saturated IMCL is linked to insulin resistance [13,14]. Studies have also shown that IMCL content varies across different muscle compartments, reflecting distinct cellular adaptations based on exercise type and muscle fiber composition [15].

In addition to lipid metabolism, there is growing clinical interest in the detection and assessment of metabolites, including choline, carnosine, taurine, myo-inositol and creatine. Creatine supplementation has been shown to increase the muscle’s creatine and phosphocreatine (PCr) content, which is essential for high-intensity exercise performance [16,17]. Choline is essential for phospholipid synthesis and triglyceride metabolism, supporting the structure and function of cell membranes and muscle cells [18]. Low choline levels are linked to various metabolic alterations, including anti-fibrotic effects [19] and muscle wasting [20], and it also contributes to muscle contraction [21]. Taurine is necessary for optimal muscle function and may help reduce muscle inflammation and enhance muscle strength [22,23]. Inositols via their major isomers (myo-inositol and D-chiro-inositol) participate in both insulin signaling and glucose metabolism [24] by influencing distinct pathways. It has also been shown that inositol is involved in excitation–contraction coupling in skeletal muscle [25].

Over the last few years, there has been steady progress in advancing in vivo magnetic resonance spectroscopy (MRS) methodology for the quantification of lipids and other metabolites in skeletal muscle [26]. One-dimensional MRS techniques result in severe overlap of lipid resonances and also hinder the detection of a wide range of metabolites at lower field strengths [27,28]. Two-dimensional (2D) localized correlated spectroscopy (L-COSY) techniques have been shown to address these issues by spreading resonances across a second dimension, which enhances the separation of spectral resonances [27,28,29]. The L-COSY techniques enable the estimation of IMCL and EMCL content in specific compartments, as well as the quantitative assessment of lipid unsaturation in vivo. However, single voxel 2D MRS techniques are limited to single muscle compartments with limited spatial coverage, and they are not efficient. Recently, using a multi-voxel 2D COSY technique, Nagarajan et al. reported differences in myolipid metabolism between type I (slow-twitch) and type II (fast-twitch) muscle fibers. They found that type I fibers have higher IMCL content compared to type II fibers in the context of obesity, and this is potentially related to IR [30,31].

Central obesity is a major player in T2DM [32,33], making it crucial to evaluate abdominal fat tissues across different compartments within the abdomen. Similarly to IMCL in skeletal muscle, triglycerides in adipose tissue and the liver have been linked to insulin resistance [34]. Non-alcoholic fatty liver disease (NAFLD), a prevalent liver condition marked by the accumulation of hepatic triglycerides and chronically elevated serum aminotransferase levels, has been reported to occur frequently in patients with T2DM [35]. There is also emerging evidence for non-alcoholic fatty pancreas disease (NAFPD) in subjects with T2DM [36,37].

In this study, a major goal was to validate lipid pools (IMCL, EMCL, unsaturation index) and more metabolites using novel accelerated five-dimensional (5D) echo-planar correlated spectroscopic imaging (EP-COSI) in the calf muscles of T2DM patients and age-matched healthy controls and to correlate the lipid and metabolite ratios with the abdominal and ectopic fat in the liver and pancreas.

## 2. Materials and Methods

### 2.1. Human Subjects

In total, 12 T2DM patients (5 males and 7 females; age = 60.3 ± 8.6 years; BMI = 28.1 ± 6.3 kg/m^2^), 9 age-matched healthy controls (AMHCs) (6 males and 3 females; age = 60.9 ± 7.8 years; BMI = 25.3 ± 2.8 kg/m^2^), and 10 young healthy controls (YHCs) (9 male and 1 female; age = 28.3 ± 1.8 years; BMI = 24.4 ± 3.3 kg/m^2^) were enrolled in the study. All participants gave informed consent in compliance with the institutional review board (IRB) at the University of California, Los Angeles.

The T2DM subjects were recruited from the UCLA Gonda Diabetic Center and from the Southern California area. The AMHCs were recruited from the UCLA community, the general community, and the family members of the patient subjects. The medical history of T2DM patients was assessed. The exclusion criteria for this study were as follows: history of cardiovascular or neuromuscular disease, HbA1c% > 9.0%, treatment with any medications that alter insulin sensitivity, documented diabetic micro- and macro-angiopathy, severe renal or hepatic disease, malignancy, or chronic inflammatory diseases such as rheumatoid arthritis. Other exclusions included subjects with claustrophobia, metallic implants such as pacemakers, defibrillators and aneurysm clips, and certain prostheses contraindicated for MRI. The inclusion criteria for healthy controls consisted of individuals in good health with sedentary lifestyles, no family history of diabetes, and women who had regular menstrual cycles.

### 2.2. Clinical and Biochemical Data

All T2DM subjects underwent blood sample collection at fasting and assessments of anthropometry via standardized methodologies. The collected data included age, gender, BMI, and levels of the aspartate aminotransferase (AST), serum creatinine (Cre), total bilirubin (TB), alanine aminotransferase (ALT), glycated hemoglobin (HbA1c), fasting glucose (Glc), alkaline phosphatase (ALP), total cholesterol (TC), triglyceride (TG), high-density lipoprotein (HDL), and low-density lipoprotein (LDL).

### 2.3. MRI/MR Spectroscopic Imaging

MRI/MRS imaging was performed on a 3T Prisma MR scanner (Siemens Medical Solutions, Erlangen, Germany) running on the VE11C platform.

#### 2.3.1. MR Spectroscopic Imaging of Calf Muscles

MR spectra of calf muscles were acquired using a 15-channel “receive” and a single-channel “transmit” knee coil in the feet-first supine position. An enhanced version of the accelerated 5D EP-COSI technique was used to obtain spectra from the right calf of each subject, with the largest diameter of the calf muscle positioned at the center of the coil [38]. The standard 5D EP-COSI sequence, which uses (90°–180°-Δt1 -90°-t2) for localization, was modified using a pair of adiabatic full passage pulses at the refocusing 180° RF pulse. The position of the volume of interest (VOI) was determined using the axial, coronal, and sagittal images to ensure coverage of the soleus, tibialis anterior, and gastrocnemius muscle regions. Interference from vascular structures and gross adipose tissue deposits was minimized by the appropriate positioning of the VOI. Anatomical MRI studies included three-plane localizer MRI (repetition time/echo time (TR/TE) = 14/5 ms, (field of view (FOV) = 45 × 45 cm^2^, matrix size = 320 × 160, one average) and T1-weighted (T1W) axial, coronal, and sagittal spin-echo MRI scans (FOV = 16 × 16 cm^2^, TR/TE = 600/2.7 ms, matrix size = 320 × 256, one average). The 5D EP-COSI acquisition was performed using a maximum echo sampling scheme with the following parameters: TR/TE = 1500/35 ms, average = 1, matrix size = 16 × 16 × 8, voxel resolution = 3.37 cm^3^, 64 Δt1 increments, 512 bipolar echo pair, acceleration factor = 8 along k_y_, k_z_ and t_1_ dimensions, and F_1_ and F_2_ bandwidths of 1250 Hz and 1190 Hz, respectively, with scan times of ~25 min. Outer volume saturation bands were applied outside the PRESS volume of interest, and manual B0 shimming was performed to reduce B0 inhomogeneity within the localized VOI, achieving a line width of approximately 12–14 Hz. A fully sampled non-water-suppressed (NWS) scan was acquired, using only the first t1 increment for eddy current correction and as a reference for coil combination, which added 30 s to the total scanning duration.

#### 2.3.2. Abdominal Fat Imaging

Abdominal MRI was performed using a 3D gradient recalled-echo (GRE) Volumetric interpolated breath-hold examination (VIBE) 6-point Dixon sequence. The acquisition parameters were voxel size = 1.187 × 1.187 × 3 mm^3^, matrix size = 320 × 240, TR = 8.85 ms, bandwidth = 1080 Hz/px, flip angle = 50^0^, and shortest possible TEs (equidistant with TE1 = 1.23 ms and an echo time shift of 1.23 ms), a GRAPPA (GeneRalized Autocalibrating Partially Parallel Acquisitions) acceleration factor of 2, and one average. Fifty-two slices were acquired between vertebral levels T12 and L5. Using the water-only and fat-only images extracted from the multi-point Dixon data, fat and water fractions were calculated via the in-line MR image reconstruction software (Siemens VE11C) as parametric maps. A gold-standard single-voxel ^1^H MRS of the liver was performed using a 15 s breath-hold, T2-corrected multi-echo spectroscopic sequence (HISTO, high-speed multiple echo acquisition), as described in [39].

### 2.4. Data Processing and Analysis

#### 2.4.1. Spectroscopic Data Processing

The acquired undersampled data were reconstructed using a group-sparsity-based compressed sensing algorithm [38]. The reconstruction was carried out for each coil individually, and the resulting coil data were combined as a sum of squares. The reconstructed data were subsequently post-processed through a series of steps, comprising scaling, spatial reordering, phase correction, resolving averages, and oversampling [38], using a custom MATLAB-based program. A detailed description of the reconstruction and postprocessing scheme can be found in [38].

For the 2D spectra recorded in the gastrocnemius, soleus, and tibialis anterior muscle regions of each subject, absolute volumes for the following metabolites were quantified using peak integration. The intramyocellular lipids were as follows: IMCL1 and IMCL2 at 5.3 ppm and 2.7 ppm and 5.3 ppm and 2.0 ppm. The extramyocellular lipids were as follows: EMCL1 and EMCL2 at 5.45 ppm and 2.85 ppm and 5.45 ppm and 2.15 ppm; choline, Ch_d (3.2 ppm, 3.2 ppm); creatine, Cr_d (3.0 ppm, 3.0 ppm); Cr_3.9 (3.9 ppm, 3.9 ppm); carnosine, Car (8.0 ppm, 8.0 ppm); taurine, Tau (3.4 ppm, 3.4 ppm); myo-inositol, mI (3.5 ppm, 3.5 ppm); triglyceryl fats, TGFR1 (5.4 ppm, 4.3 ppm) and TGFR2 (4.3 ppm, 5.4 ppm); unsaturated fatty acids, Unsat_CH = CH, FAT_5.4 (5.4 ppm, 5.4 ppm); and poly-methylene protons of fat, FAT_1.4 (1.4 ppm, 1.4 ppm). We identified the diagonal peak at [3.5 ppm, 3.5ppm] as myo-inositol based on earlier ex vivo magic angle spinning spectroscopy of muscle tissues [40]. The peak assignment and integration were performed as described by Srikanthan et al. [41].

Since the total muscle creatine (Cr_d) content remains stable in T2DM, it was used as an internal reference for the normalization of the other peaks in the muscle spectrum. The peak ratios were determined by dividing the absolute peak volume of each metabolite by the volume of the Cr_d peak. The unsaturation indices (UIs) of IMCL and EMCL were estimated using the ratio of the cross-peaks of IMCL1/IMCL2 and EMCL1/EMCL2, respectively [27,28,41]. The average EMCL (Avg EMCL) and IMCL (Avg IMCL) were calculated from EMCL1, EMCL2, and IMCL1 and IMCL2, respectively.

#### 2.4.2. Abdominal Fat Segmentation

We used an image analysis software slice-O-matic (Ver. 5.0, Tomovision, Canada) to quantify subcutaneous adipose tissues (SATs), visceral adipose tissues (VATs), and total abdominal fat (TAT). A single trained observer (MKS) guided by an experienced radiologist (ERF/SR) performed the image analysis. The VAT and SAT areas (cm^2^) were automatically calculated by summing the pixels corresponding to each tissue region in each slice and then multiplying via the pixel surface area. Finally, the tissue volume (cm^3^) for each slice was determined by multiplying the tissue area (cm^2^) by the thickness of the slice.

#### 2.4.3. Liver and Pancreatic Fat

Hepatic fat fraction (HFF) was measured by ROIs selected in homogeneous sections of the liver. To calculate the pancreatic fat fraction (PFF), ROIs were placed in the head (caput, PFFHead) and body/tail (corpus + cauda, PFFBody + Tail) of the pancreas. Pancreatic regions were segmented into two parts: head and body/tail. To prevent segmentation errors, PFF was not calculated separately for the body and tail. The mean pixel value obtained from the software (*SliceOmatic* (Ver. 5.0), Tomovision, Magog, QC, Canada) was scaled down by a factor of 1000 to compensate for the scaling applied during image acquisition on the MRI scanner.

For HISTO, the fat fraction values (HFF_MRS) and R2 (1/T2) relaxation rate of water (R2_WAT) were calculated using the vendor-provided software package (Siemens VE11C), as described in [39].

### 2.5. Statistical Analyses

All statistical analyses were conducted using SPSS software (V25.0, IBM, Chicago, IL, USA). A *p*-value of less than 0.1 was considered statistically significant. The independent sample *t*-tests were performed to assess differences in lipids and metabolite ratios between T2DM and AMHC and between the AMHC and YHC groups. Pearson’s correlation was used to determine the association between MRS lipids/metabolites and MRI-based fat estimates and also between MRI/MRS parameters and blood chemistry in the T2DM and AMHC groups.

## 3. Results

**Figure 1:**
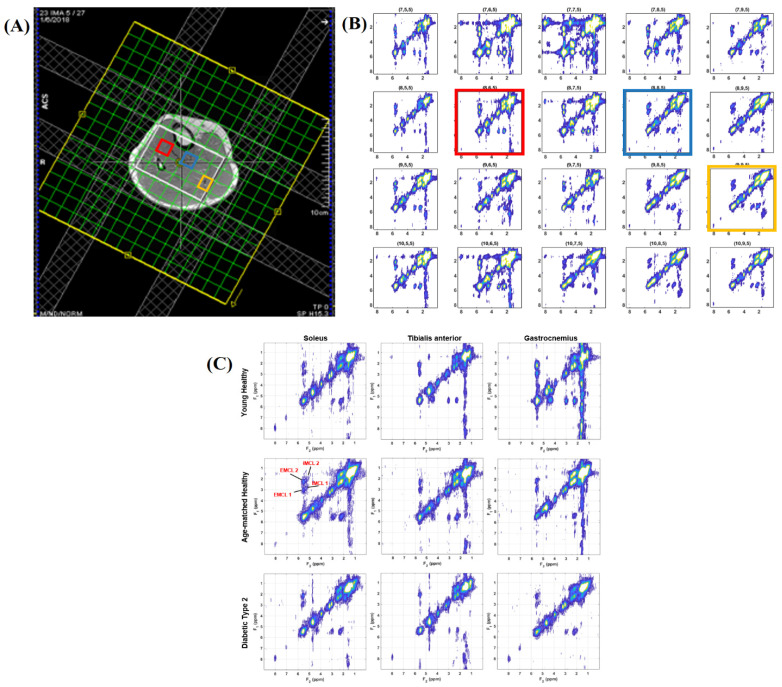
(**A**) Axial positioning of the volume of interest (VOI) in the calf muscle of a 55-year-old patient with type 2 diabetes. (**B**) Multi-voxel COSY spectra from a 4 × 5 region encompassing the tibialis anterior (red), soleus (blue), and gastrocnemius (yellow) muscles. (**C**) Extracted spectra from the soleus, tibialis anterior, and gastrocnemius calf muscles in one young, age-matched healthy control, and a T2DM patient. Notably, the diagonal peak of carnosine (8 ppm) was observed in the soleus muscles of all three individuals. The diagonal creatine peak at 3.9 ppm appears as a doublet in all tibialis anterior muscles, while it remains a single peak in the soleus. IMCL and EMCL are marked in red text.

The 5D EP-COSI technique allows wider coverage of the calf muscle, differentiating spectral characteristics of muscle metabolites and lipids in various muscle compartments, soleus (SOL), tibialis anterior (TA), and gastrocnemius (GAS) (Figure 1A). Differences between these regions are shown in Figure 1B, where the diagonal peak of carnosine (8 ppm) is present in all soleus muscles. In contrast, the diagonal peak of creatine at 3.9 ppm appears as a doublet in the tibialis anterior and gastrocnemius muscle regions, while it remains a single peak in the soleus. The doublet is a result of residual dipolar coupling in the type 2 muscle fibers. Extracted 2D COSY spectra are shown from the SOL, TA, and GAS muscle regions in T2DM and age-matched HC and young HC subjects.

The mean age was similar between the T2DM and AMHC groups (60.25 ± 8.59 vs. 60.89 ± 7.82 years). The subjects in the T2DM group had a higher BMI compared to those in the AMHC and YHC groups (28.08 ± 6.35 vs. 25.30 ± 2.83 vs. 24.41 ± 3.28 kg/m^2^), but they were not statistically significant.

### 3.1. Calf Muscle MRS (YHC vs. AMHC)

The choline levels in gastrocnemius and soleus were significantly lower in YHC compared to AMHC groups (Table 1). However, TA choline in YHC was more than 2-fold higher than in the AMHC group. EMCL1 and IMCL2 in the soleus were lower in YHC compared to AMHC. However, the unsaturation index of EMCL in TA and IMCL in GAS was higher in YHC than in AMHC. Carnosine levels in the soleus and taurine levels in the TA were higher, and myoinositol in GAS and TA were lower in YHC than in AMHC. YHC had lower levels of creatine in GAS and higher levels of creatine in TA compared to AMHC. The TGFR1 was 5-fold higher in the YHC group compared to AMHC.

### 3.2. Calf Muscle MRS and Abdominal MRI (T2DM vs. AMHC)

**Figure 2:**
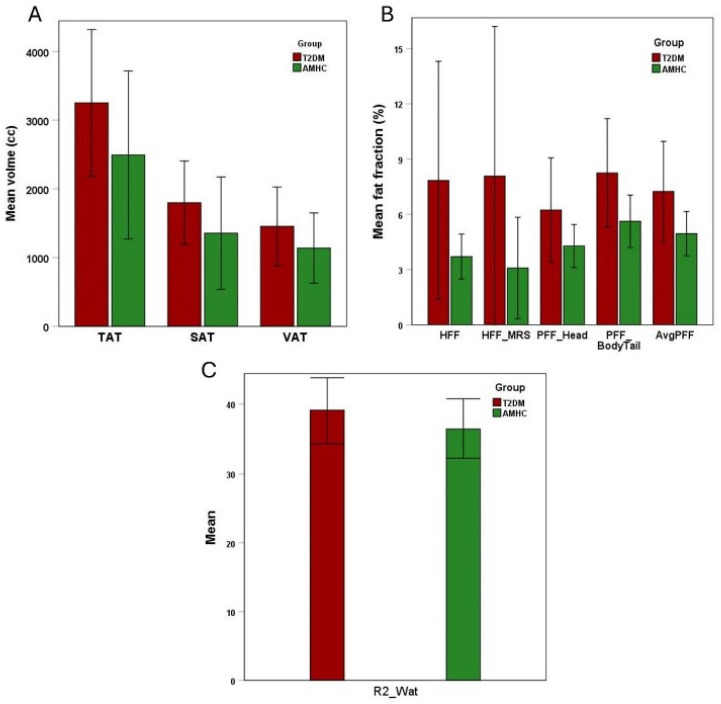
MRI/MRS-based hepatic, abdominal, and pancreatic fat in T2DM and AMHC groups. (**A**) Mean volumes of TAT, SAT and VAT; (**B**) Mean Fat fractions of HFF, HFF_MRS, PFF_Head, PFF_Body Tail and AvgPFF; (**C**) Mean R2_Wat values.

The T2DM subjects had more than 2-fold higher (1.13 ± 0.91 vs. 0.58 ± 0.22) levels of choline in TA muscles compared with the AMHC group (Table 1). Myoinositol in the soleus muscle of T2DM was higher (0.07 ± 0.02 vs. 0.05 ± 0.02) than in the AMHC group. TGFR2 in TA muscles was significantly lower (0.02 ± 0.01 vs. 0.03 ± 0.01) in T2DM compared with AMHC subjects. The unsaturation index of IMCL and EMCL in GAS, SOL, and TA muscles was lower in T2DM than in AMHC, but it was not statistically significant. Similarly, creatine levels were higher in T2DM compared with AMHC, but they did not reach statistical significance. Fat in the liver, pancreas, and abdomen was higher in T2DM subjects compared with AMHC subjects (Figure 2). Figure 2A shows mean volumes of TAT, SAT and VAT; Figure 2B shows mean Fat fractions of HFF, HFF_MRS, PFF_Head, PFF_Body Tail and AvgPFF; and Figure 2C shows mean R2_Wat values.

### 3.3. Association Between MRS and MRI Fat Measures in T2DM

**Figure 3:**
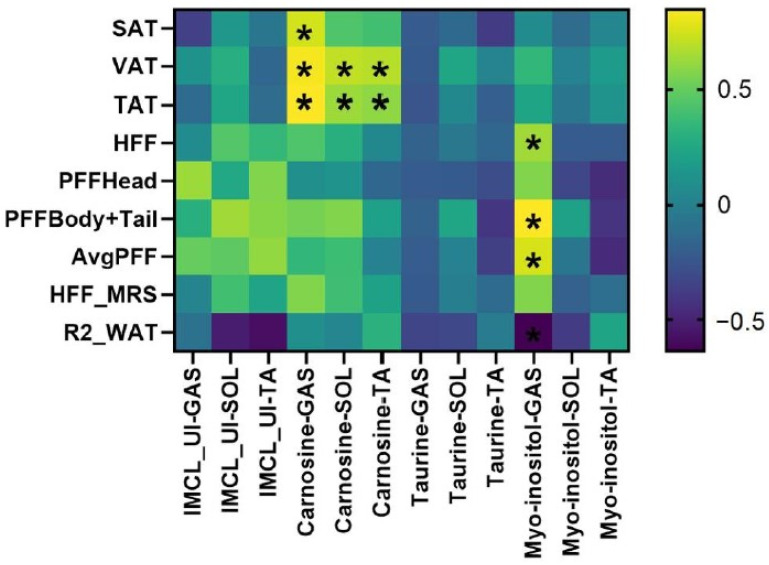
Heatmap showing the Pearson correlation (r) between MRI-based liver, muscle, abdominal, and pancreatic fat and MRS-based lipids/metabolites in the T2DM group. Statistically significant (*p* < 0.1) associations are highlighted via *.

Figure 3 presents the Pearson correlations of the IMCL unsaturation index and carnosine, taurine, and myoinositol in gastrocnemius, soleus, and tibialis anterior muscles with estimates of liver, pancreas, and abdominal fat in the T2DM subjects. Carnosine in all the muscle groups was significantly associated with VAT and TAT, whereas Carnosine in only GAS was associated with SAT. Myo-inositol in GAS was significantly associated with liver and pancreatic fat fraction.

Table 2 lists all lipids, metabolites, and fat estimates that were significantly associated with blood markers. The magnitude of all these associations was high (>0.5). To highlight a few, the IMCL unsaturation index in TA was negatively associated with HbA1c and CHOLDLCAL. SAT volume was associated with Glc and CHOHDL, whereas VAT was significantly associated with ALT. Liver fat fractions estimated via both MRI and MRS were significantly associated with ALT.

## 4. Discussion

There is a large interest in understanding ectopic fat accumulation in progressive metabolic diseases [42]. The quantification of IMCL and EMCL during aging and diabetes is of large clinical interest due to its association with inflammation and muscle loss [43]. The contribution of individual muscle compartments with different combinations of fiber types and their association with skeletal muscle metabolism is not known. In that context, there is a large interest in the implementation of advanced MRS methodology for investigating various muscle compartments.

Even though conventional one-dimensional MRSI techniques can provide information on the distribution of IMCL / EMCL in various compartments [44], it is a challenge to investigate the composition of the lipids at clinical field strengths. In this context, there is also clinical interest in understanding the role of saturated and unsaturated lipids within these lipid pools in the development of insulin resistance [14]. To address this issue, we earlier implemented single-voxel-based localized 2D MRS techniques to separate the overlapping lipid pools and also resolve various metabolites [27,28]. However, single-voxel-based techniques have limited spatial coverage involving acquisition from one muscle compartment. In this study, we implemented spatially resolved 2D MRS via accelerated 5D EP-COSI along with body composition imaging to estimate lipids and metabolites in young and old healthy subjects along with diabetic subjects. We were able to measure the lipid composition within IMCL and EMCL along with various metabolites, including choline, carnosine, and taurine. Recent work has shown that circulating taurine deficiency may be a driver of aging [23]. In that context, in vivo detection of taurine in skeletal muscle will open new possibilities for trials with taurine. We also identified the diagonal peak at [3.5 ppm, 3.5 ppm] as myo-inositol based on the earlier ex vivo HRMAS of muscle tissues.

We also evaluated the body composition, including subcutaneous fat (SAT), visceral fat (VAT), hepatic fat fraction (HFF), and pancreatic fat fraction (PFF), in age-matched healthy controls and diabetic subjects. We observed the lower unsaturation of IMCL and EMCL in GAS, SOL, and TA in T2DM compared to the AHMC group, although this was not statistically significant. It is known that the fatty acid synthase enzymes can become defective in diabetes [12]. We also observed higher choline levels in both T2DM and AHMC groups compared to YHC. Choline is an essential micronutrient and plays an important role in several metabolic pathways. A low concentration of Choline is also associated with muscle wasting [19]. We also observed higher levels of myoinositol in the T2DM group. It is known that inositols are involved in excitation–contraction coupling in skeletal muscle [25]. The higher choline and inositol levels in the T2DM group might be due to compensatory mechanisms that protect muscle loss.

The correlation analysis identified an association between the IMCL unsaturation index and abdominal fat content. Given the strong link between IMCL and insulin sensitivity, these findings help elucidate the role of dysfunctional adipose tissue in T2DM. Both EP-COSI and Dixon MRI demonstrated consistent trends in distinguishing T2DM patients from healthy controls. However, due to the small sample size in this study, no significant differences were observed between the two groups. In the AMHC, visceral fat was found to be correlated with IMCL, a relationship also reported in previous studies of lean and obese children [45]. This correlation was not observed in the T2DM group.

In a recent study by Sarma et al., adipose tissue distribution included SAT, VAT, HFF, and PFF in T2DM patients, and these findings were compared with nondiabetic age-matched adults and young healthy subjects [39]. A strong correlation was observed between hepatic fat fraction estimates from 6-point Dixon MRI and HISTO-MRS. The increased SAT and VAT further support the link between central obesity and the progression of T2DM, promoting insulin resistance. VAT was higher in the T2DM group compared to both AMHC and YHC, with the greatest difference observed between T2DM and YHC. Additionally, T2DM showed nearly equal percentages of SAT and VAT, indicating that VAT accumulation may play a significant role in the development of T2DM. T2DM patients exhibited significantly higher hepatic fat content compared to both AHMC and YHC subjects, suggesting that T2DM is linked to increased fat accumulation in the liver. The HFF in individuals with T2DM was found to be 44.6% higher compared to age-matched healthy controls and 64.4% higher compared to young healthy controls.

## 5. Limitation

A limitation of this study is its cross-sectional design and the small sample size. Due to the small sample size, we could not perform Bonferroni corrections for correlations. Although we observed significant differences in VAT, HFF, and PFF in T2DM patients compared to AMHC, larger cohort studies are needed to determine whether these changes play a key role in the development of insulin resistance and impaired glucose tolerance and the progression to T2DM. However, this preliminary study using 5D EPCOSI in conjunction with body composition imaging reveals important differences between T2DM and healthy subjects.

## Figures and Tables

**Table 1:** Lipids and metabolite ratios within soleus, tibialis anterior, and gastrocnemius muscles in T2DM, AMHC, and YHC groups.

Metabolites	Muscle	T2DM Mean ± SD	AMHC Mean ± SD	YHC Mean ± SD	*p* Value T2DM vs. AMHC	*p* Value AMHC vs. YHC
Ch_d	GAS SOL TA	1.15 ± 0.28 1.00 ± 0.32 1.13 ± 0.91	1.02 ± 0.13 1.21 ± 0.54 0.58 ± 0.22	0.63 ± 0.36 0.43 ± 0.30 1.26 ± 1.01	0.274 0.280 **0.097**	**0.013** **0.001** **0.066**
EMCL1	GAS SOL TA	0.07 ± 0.05 0.11 ± 0.06 0.12 ± 0.07	0.07 ± 0.05 0.08 ± 0.06 0.12 ± 0.07	0.09 ± 0.08 0.04 ± 0.02 0.15 ± 0.14	0.910 0.368 0.994	0.524 **0.049** 0.660
EMCL2	GAS SOL TA	0.12 ± 0.09 0.15 ± 0.09 0.20 ± 0.12	0.11 ± 0.05 0.14 ± 0.09 0.21 ± 0.13	0.16 ± 0.12 0.10 ± 0.07 0.28 ± 0.26	0.815 0.735 0.926	0.296 0.294 0.497
Avg EMCL	GAS SOL TA	0.09 ± 0.07 0.13 ± 0.07 0.16 ± 0.08	0.09 ± 0.05 0.11 ± 0.07 0.17 ± 0.10	0.12 ± 0.09 0.07 ± 0.05 0.22 ± 0.20	0.847 0.549 0.940	0.367 0.141 0.550
EMCL UI	GAS SOL TA	1.68 ± 0.68 1.59 ± 0.91 1.36 ± 0.41	1.72 ± 0.52 1.89 ± 0.85 1.51 ± 0.53	1.87 ± 0.48 2.49 ± 0.75 2.00 ± 0.59	0.895 0.452 0.478	0.580 0.119 **0.076**
IMCL1	GAS SOL TA	0.02 ± 0.02 0.02 ± 0.02 0.01 ± 0.01	0.01 ± 0.01 0.02 ± 0.01 0.01 ± 0.01	0.01 ± 0.00 0.01 ± 0.01 0.02 ± 0.02	0.410 0.248 0.817	0.111 0.106 0.191
IMCL2	GAS SOL TA	0.02 ± 0.03 0.04 ± 0.03 0.02 ± 0.01	0.01 ± 0.00 0.02 ± 0.01 0.02 ± 0.01	0.02 ± 0.01 0.02 ± 0.01 0.02 ± 0.01	0.385 0.275 0.888	0.591 **0.027** 0.172
Avg IMCL	GAS SOL TA	0.02 ± 0.02 0.03 ± 0.03 0.02 ± 0.01	0.01 ± 0.00 0.02 ± 0.01 0.01 ± 0.01	0.01 ± 0.01 0.01 ± 0.01 0.02 ± 0.01	0.386 0.252 0.832	0.835 **0.037** 0.121
IMCL UI	GAS SOL TA	1.31 ± 0.46 1.52 ± 0.53 1.48 ± 0.68	1.34 ± 0.49 1.79 ± 0.81 1.66 ± 1.18	2.16 ± 1.10 1.85 ± 0.66 1.76 ± 1.23	0.902 0.369 0.684	**0.074** 0.856 0.859
Car	GAS SOL TA	0.01 ± 0.01 0.01 ± 0.01 0.01 ± 0.02	0.01 ± 0.01 0.02 ± 0.01 0.01 ± 0.02	0.01 ± 0.01 0.02 ± 0.01 0.02 ± 0.01	0.894 0.597 0.938	0.345 **0.077** 0.300
Tau	GAS SOL TA	0.19 ± 0.24 0.16 ± 0.09 0.33 ± 0.79	0.13 ± 0.06 0.14 ± 0.06 0.11 ± 0.11	0.10 ± 0.09 0.16 ± 0.07 0.34 ± 0.33	0.538 0.570 0.430	0.371 0.451 **0.061**
mI	GAS SOL TA	0.06 ± 0.03 0.07 ± 0.02 0.10 ± 0.10	0.05 ± 0.02 0.05 ± 0.02 0.06 ± 0.05	0.03 ± 0.02 0.07 ± 0.07 0.25 ± 0.26	0.257 **0.067** 0.336	**0.009** 0.501 **0.049**
Cr_3.9	GAS SOL TA	0.37 ± 0.13 0.29 ± 0.07 0.46 ± 0.48	0.30 ± 0.08 0.29 ± 0.06 0.27 ± 0.23	0.21 ± 0.10 0.43 ± 0.30 0.82 ± 0.64	0.233 0.941 0.292	**0.060** 0.170 **0.026**
TGFR1	GAS SOL TA	0.03 ± 0.02 0.07 ± 0.06 0.14 ± 0.09	0.03 ± 0.03 0.11 ± 0.16 0.22 ± 0.18	0.16 ± 0.17 0.06 ± 0.08 0.29 ± 0.35	0.989 0.400 0.211	**0.062** 0.407 0.633
TGFR2	GAS SOL TA	0.02 ± 0.02 0.01 ± 0.01 0.02 ± 0.01	0.02 ± 0.01 0.01 ± 0.01 0.03 ± 0.01	0.03 ± 0.03 0.02 ± 0.02 0.04 ± 0.03	0.941 0.622 **0.030**	0.142 0.754 0.252
FAT_1.4	GAS SOL TA	11.85 ± 5.62 15.11 ± 6.69 15.77 ± 6.15	11.10 ± 5.36 10.76 ± 4.35 18.54 ± 5.85	9.63 ± 5.45 11.11 ± 9.34 21.84 ± 11.39	0.790 0.107 0.310	0.594 0.920 0.447
FAT_5.4	GAS SOL TA	0.86 ± 0.46 1.17 ± 0.45 1.22 ± 0.44	0.73 ± 0.35 0.96 ± 0.41 1.81 ± 0.80		0.563 0.282 **0.043**	

Statistically significant (*p* < 0.1) differences are highlighted in bold.

**Table 2:** Pearson correlation of lipid/metabolites and body fat with blood chemistry in the T2DM group. HbA1c: Hemoglobin A1C; Cre: creatinine; Glc: glucose; AST: aspartate aminotransferase; ALT: alanine aminotransferase; CHO: cholesterol; CHOLDL: low-density lipoprotein cholesterol; CHOHDL: high-density lipoprotein cholesterol; NHDLCHO: non-high-density lipoprotein cholesterol; AP: alkaline phosphatase; TB: total bilirubin.

Blood Chemistry	Lipid/Metabolites and Body Fat	Correlation Coefficient (r), *p* Value
HbA1c	EMCL2 (TA) avgEMCL (TA) EMCLUI (SOL) IMCLUI (TA) Car (SOL) Car (TA) Cr_3.9 (SOL) TGFR1 (TA) R2_WAT	0.73, *p* = 0.039 0.70, *p* = 0.055 0.64, *p* = 0.065 −0.63, *p* = 0.094 0.59, *p* = 0.098 0.65, *p* = 0.056 0.60, *p* = 0.088 0.77, *p* = 0.015 0.62, *p* = 0.073
Triglycerides	Ch_d (SOL)	0.59, *p* = 0.096
CHOLDL	Ch_d (GAS) IMCLUI (TA) mI (GAS) Cr_3.9 (GAS) R2_WAT	−0.62, *p* = 0.098 −0.74, *p* = 0.037 −0.68, *p* = 0.062 −0.72, *p* = 0.045 0.94, *p* = 0.0001
Cre	EMCLUI (GAS) EMCLUI (SOL) avgIMCL (GAS)	0.77, *p* = 0.071 −0.76, *p* = 0.048 −0.89, *p* = 0.044
Glc	Ch_d (SOL) EMCL1 (TA) IMCLUI (GAS) SAT	0.70, *p* = 0.082 0.75, *p* = 0.085 0.78, *p* = 0.067 −0.71, *p* = 0.075
CHO	mI (SOL) R2_WAT	−0.79, *p* = 0.061 0.95, *p* = 0.004
CHOHDL	EMCLUI (TA) Cr_3.9 (SOL) TGFR1 (SOL) SAT TAT	0.80, *p* = 0.058 −0.96, *p* = 0.003 0.83, *p* = 0.043 0.83, *p* = 0.042 0.84, *p* = 0.037
NHDLCHO	EMCL2 (TA) R2_WAT	0.85, *p* = 0.068 0.97, *p* = 0.001
AST	EMCL1 (GAS) EMCL1 (SOL) EMCL2 (GAS) avgEMCL (GAS) IMCLUI (TA) Car (SOL) Tau (GAS) TGFR1 (GAS) FAT_1.4 (SOL)	−0.91, *p* = 0.095 −0.82, *p* = 0.09 −0.94, *p* = 0.063 −0.95, *p* = 0.055 0.86, *p* = 0.06 0.90, *p* = 0.036 0.93, *p* = 0.066 −0.92, *p* = 0.084 −0.89, *p* = 0.042
ALT	EMCLUI (GAS) IMCL2 (TA) VAT HFF HFF_MRS	0.93, *p* = 0.066 −0.97, *p* = 0.033 0.96, *p* = 0.011 0.88, *p* = 0.049 0.90, *p* = 0.038
AP	TGFR1 (TA) R2_WAT	0.92, *p* = 0.028 0.91, *p* = 0.034
TB	EMCLUI (SOL) IMCL2 (GAS) Cr_3.9 (SOL)	−0.87, *p* = 0.057 −0.99, *p* = 0.083 −0.84, *p* = 0.076

## Data Availability

The datasets used and/or analyzed during the current study are available from the corresponding author upon reasonable request.
